# eHealth in Care Coordination for Older Adults Living at Home: Scoping Review

**DOI:** 10.2196/39584

**Published:** 2022-10-18

**Authors:** Hilde Marie Hunsbedt Fjellså, Anne Marie Lunde Husebø, Marianne Storm

**Affiliations:** 1 Department of Public Health Faculty of Health Sciences University of Stavanger Stavanger Norway; 2 Research Group of Nursing and Health Sciences Stavanger University Hospital Stavanger Norway; 3 Faculty of Health Sciences and Social Care Molde University College Molde Norway

**Keywords:** eHealth, care coordination, older adults, primary health care, mobile phone

## Abstract

**Background:**

The population of older adults is projected to increase, potentially resulting in more older adults living with chronic illnesses or multimorbidity. Living with chronic illnesses increases the need for coordinated health care services. Older adults want to manage their illnesses themselves, and many are positive about using eHealth for care coordination (CC). CC can help older adults navigate the health care system and improve information sharing.

**Objective:**

This study aimed to map the research literature on eHealth used in CC for older adults living at home. This study assessed CC activities, outcomes, and factors influencing the use of eHealth in CC reported by older adults and health care professionals.

**Methods:**

We used a scoping review methodology. We searched four databases—MEDLINE, CINAHL, Academic Scoping Premier, and Scopus—from 2009 to 2021 for research articles. We screened 630 records using the inclusion criteria (older adults aged >65 years, primary health care setting, description of an eHealth program or intervention or measure or experiences with the use of eHealth, and inclusion of CC or relevant activities as described in the Care Coordination Atlas). The analysis of the included articles consisted of both a descriptive and thematic analysis.

**Results:**

A total of 16 studies were included in this scoping review. Of these 16 studies, 12 (75%) had a quantitative design, and the samples of the included studies varied in size. The categories of eHealth used for CC among older adults living at home were electronic health records and patient portals, telehealth monitoring solutions, and telephone only. The CC activity communication was evident in all studies (16/16, 100%). The results on patient- and system-level outcomes were mixed; however, most studies (7/16, 44%) reported improved mental and physical health and reduced rehospitalization and hospital admission rates. Observing changes in patients’ health was a facilitator for health care professionals using eHealth in CC. When using eHealth in CC, available support to the patient, personal continuity, and a sense of security and safety were facilitators for older adults. Individual characteristics and lack of experience, confidence, and knowledge were barriers to older adults’ use of eHealth. Health care professionals reported barriers such as increased workload and hampered communication.

**Conclusions:**

We mapped the research literature on eHealth-enabled CC for older adults living at home. We did not map the gray literature as we aimed to map the research literature (peer-reviewed research articles published in academic journals). The study results showed that using eHealth to coordinate care for older adults who live at home is promising. To ensure the successful use of eHealth in CC, we recommend customized eHealth-enabled health care services for older adults, including individualized education and support.

## Introduction

### Background

It is estimated that the population of older adults aged >65 years will double between 2010 and 2050, and over half of them are expected to live with multimorbidity [1-4]. Aging causes older adults to live with potentially both frailty and chronic illnesses, both affecting their health trajectory [5]. Furthermore, noncommunicable diseases (cardiovascular diseases, cancers, chronic respiratory diseases, and diabetes) are a global health challenge [6]. The World Health Organization calls for better management of noncommunicable diseases and mental health conditions in primary health care, especially among older adults [7]. Living with chronic illness or multimorbidity often results in fragmented health care services and a lack of information sharing among members of the health care team and between health care professionals and patients [8-10]. Care coordination (CC) can reduce system fragmentation, help patients navigate the health care system, and improve information sharing [11]. In this scoping review, we understand CC according to the definition by McDonald et al [12]: “The deliberate organization of patient care activities between two or more participants (including the patient) involved in a patient’s care to facilitate the appropriate delivery of health care services [...].” We use the term *health care professionals*, which includes nurses working in both specialist and primary care, physicians, or general and specialist practitioners [13].

Health ITs (HITs), electronic health records (EHRs), and patient portals are important tools for CC that enable health care professionals and patients to share, access, and manage information [14,15]. Other types of eHealth include health applications, telehealth, contact through telephone use, and other medical devices such as sensor technology [16]. McDonald et al [11] describe HIT as an enabler of coordination as it makes it possible to exchange and share information and communicate among health care professionals as well as with patients [16].

Previous research has shown that many older adults want to manage their illnesses themselves [17-20], and many are positive about the use of eHealth [18]. This aligns with the expectation of treating and caring for older adults with multimorbidity or chronic illnesses in their homes [21-24]. An explorative qualitative study of primary health care professionals and older adults living at home points out that electronic care plans can improve primary care by ensuring accessible information for patients, next of kin, or health care professionals [25]. Husebø and Storm [26] reported that the use of video communication in web-based home visits to older adults can facilitate continuous and coordinated care between the patient and health care professionals. Improved information flow among health care professionals in the primary care and specialist health care services can be associated with fewer emergency department (ED) visits and reduce the likelihood of outpatient visits among older adults [27]. Kooij et al [28] conducted a systematic review of HIT interventions to support shared care for patients with chronic illnesses and reported that EHRs resulted in fewer rehospitalizations and more visits to primary care physicians.

Peterson et al [29] conducted a systematic scoping review of 37 CC frameworks and identified a need to increase the use of theoretical frameworks when assessing care initiatives, especially in a primary care setting. Peterson et al [29] pointed out that the definition of CC by McDonald et al [11] is the most cited. The Care Coordination Atlas framework focuses on organizing and evaluating measures [29]. McDonald et al [11] organized CC measures into activities that enhance CC. These activities are directed at health care professionals and include facilitating information exchange and communication, facilitating transitions, assessing the patient’s needs and goals, creating a proactive plan of care, monitoring, following up and responding to change, supporting self-management goals, linking to community resources, and aligning resources with patient and population needs. The framework can adapt to the developing CC field and is especially relevant for CC in the primary health care setting [11]. The framework also suggests validated measures for each of the activities [11,29]. Thus, the Care Coordination Atlas was used in this scoping review to identify and report CC activities when using eHealth in CC for older adults.

### Objectives

A limited amount of research has focused on eHealth to support CC in older adults [30]. There is a need to gain more knowledge on how best to support older adults using eHealth [31] and particularly to examine eHealth in CC for older adults living at home [30]. This scoping review mapped the research literature (peer-reviewed research articles published in academic journals) to explore the use of eHealth in CC for older adults. The research questions that guided our review were as follows: (1) What categories of eHealth can be identified in the research literature and how do the CC activities relate to the eHealth categories? (2) What are the patient and health care use outcomes associated with the use of eHealth in CC? (3) What factors influencing the use of eHealth in CC are reported by older adults and health care professionals?

## Methods

### Scoping Review Methodology

We followed the Arksey and O’Malley scoping studies framework [32]: (1) identifying the research questions; (2) identifying relevant studies; (3) selecting the studies; (4) charting the data; and (5) collating, summarizing, and reporting results. In addition, the PRISMA-ScR (Preferred Reporting Items for Systematic Reviews and Meta-Analyses extension for Scoping Reviews) checklist and explanation developed by Tricco et al [33] were used as a reporting tool.

### Identifying Relevant Studies (Databases and Search Terms)

The search was conducted in the MEDLINE, CINAHL, Academic Search Premier, and Scopus databases and included research articles published between 2009 and 2021. The last search was conducted in December 2021 by HMHF in collaboration with a university librarian. Search terms related to CC (*coordinated care, integrated care, integrated health, care management, patient care management, case management, care transition, continuity of care, care planning, continuum of care,* and *shared care*), eHealth (*telecare, telehealth, telemedicine, remote consultation, assistive technology, electronic health record, information communication technology,* and *mhealth*), home care (*home care services, home nursing, community-dwelling, independent living, home based care, community health services, municipal health services, primary health care,* and *general practitioner*), and older patients (*elderly, aged, older person, elderly and chronic illness, elderly and multimorbidity, older adult,* and *frail elderly*) were used. In addition, Medical Subject Headings and thesaurus terms were used when possible (see Multimedia Appendix 1 for all search terms and an example of a search).

### Selection of Studies

The selected studies were included based on a 2-step iterative process. First, we developed and tested a set of preliminary eligibility criteria, which we used to screen the titles. All authors met to discuss the preliminary eligibility criteria and did some final modifications (Textbox 1). Second, we tested the final eligibility criteria on 20 titles and abstracts and found them fitting. The final eligibility criteria (Textbox 1) were used to screen all titles and abstracts in collaboration with all authors. HMHF screened all articles (both titles and abstracts), and AMLH and MS screened 30 titles and abstracts each. All full-text articles were screened by HMHF. The 3 authors screened the same 11 full-text articles. The included articles were read by all authors.

**Final eligibility criteria.**

**Inclusion criteria**

- Older adults aged >65 years
- Primary health care setting; older adults living in their own home
- Describing an eHealth program or measure or intervention or experiences with the use of eHealth
- Including care coordination or relevant activities as described in the Care Coordination Atlas
- Published after 2009
- Reported in English
- Peer-reviewed when possible to choose a limitation in the database

**Exclusion criteria**

- Older adults aged <65 years, next of kin, informal caregivers, and studies including different age groups when it was not possible to extract data on those aged >65 years
- Older adults living in nursing homes or who were in a hospital
- Studies with a primary focus on cost-effectiveness
- Books, book chapters, literature reviews, study protocols, conference and poster abstracts and papers, editorials, and discussion papers

The EPPI-Reviewer (version 4; EPPI-Centre) software [34] was used in the screening process. All 3 authors met and discussed the inclusion and exclusion of records according to the eligibility criteria. Disagreements regarding inclusion or exclusion were resolved through discussions between the authors. Agreement on inclusion was reached for all articles.

### Charting the Data

Descriptive data were charted from each article according to the following: authors; country of origin; study population (age group and number of participants); and type of eHealth program, intervention, measure, or experience with eHealth. For the articles that described patient or health care use outcomes, we extracted and charted these results when applicable. Data relevant to CC activities and factors influencing the use of eHealth in CC were also extracted. Data charting was conducted by HMHF with input from AMLH and MS.

### Collating, Summarizing, and Reporting the Results

We prepared a descriptive summary of the study characteristics (country of origin, methods used, overview of included participants, and year of publication). We were inspired by a thematic analysis to thematically organize and present the study results [35]. The first author conducted an inductive analysis to identify codes of eHealth tools or solutions described in the articles, which were classified into 3 eHealth categories. Arksey and O’Malley [32] suggest using a theoretical framework to summarize and describe variables. Hence, we conducted a deductive thematic analysis to identify CC activities in the eHealth categories. HMHF searched for and documented relevant CC activities in the included studies. To identify patient and health care use outcomes and factors influencing the use of eHealth in CC, we used an inductive thematic analysis. Outcomes were coded and categorized into patient-level and system-level outcomes. When analyzing factors influencing the use of eHealth, HMHF identified codes, which were categorized into facilitators of and barriers to the use of eHealth in CC. HMHF led the analysis process. The codes and identified categories were discussed with MS in 6 analysis meetings, and AMLH participated in 2 meetings.

## Results

### Overview

**Figure 1:**
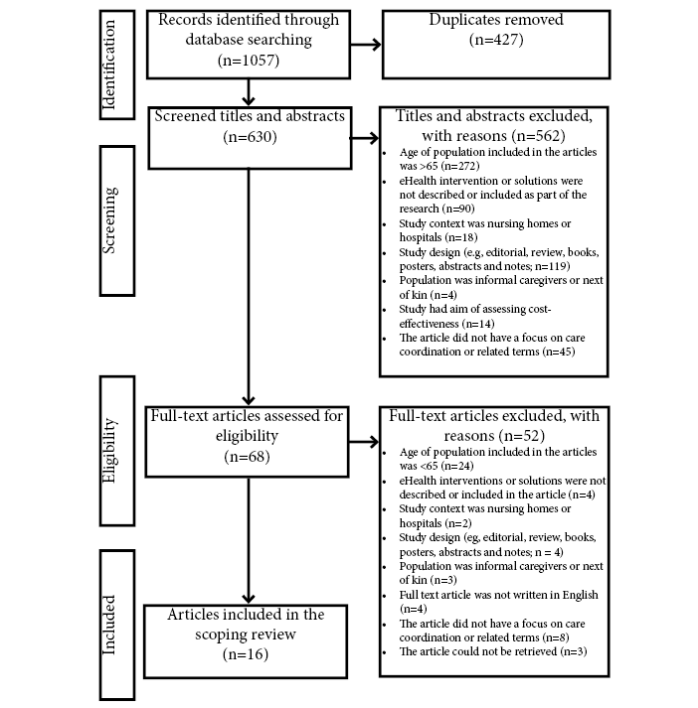
PRISMA (Preferred Reporting Items for Systematic Reviews and Meta-Analyses) flow diagram.

The study selection process is illustrated in Figure 1 [36]. A total of 1057 records were identified; after duplicates were removed, we screened the titles and abstracts of 630 (59.6%) records. A total of 89.2% (562/630) of titles and abstracts and 76% (52/68) of full-text articles were excluded, and the reasons are documented in the PRISMA (Preferred Reporting Items for Systematic Reviews and Meta-Analyses) flow diagram in Figure 1. The main reason for exclusion was that the study population did not meet the age criterion (>65 years). Another frequent reason for exclusion was that the study did not describe an eHealth intervention or experience with eHealth. A total of 68 articles were assessed in full text for eligibility, of which 16 (24%) were included in this scoping review.

### Study Characteristics

Of the 16 included articles, 4 (25%) were from 2 research studies and had the same first authors: Makai et al [37,38] and Gellis et al [39,40]. The study sample sizes varied. The smallest sample size was reported in the study by Gokalp et al [41] (N=36). A research article presenting a randomized controlled trial (RCT) had the largest sample size (N=3661). See an overview of the study designs and participant characteristics in Table 1.

**Table 1:** Overview of study designs and participant characteristics (N=16).

Authors, country of origin	Type of study design	Participant characteristics	Perspectives represented in the study
Sheeran et al [42], United States	Quantitative pilot study	55 participants 48 patients with depression 7 health care professionals	Patients Health care professionals
Logue and Effken [43], United States	Quantitative pilot study	38 patients with chronic illnesses	Patients
Gokalp et al [41], United Kingdom	Quantitative pilot study or technical review	36 patients; frail older adults with at least one chronic disease Service team including health care professionals (number not specified)	Patients Health care professionals
Lewis et al [44], Ireland	Quantitative observational study	54 patients; frail older adults with comorbidities such as dementia, cardiovascular disease, hypertension, cerebral vascular disease, or COPD^a^	Patients
De Jong et al [45], Netherlands	Quantitative observational study	96 patients with a dementia diagnosis	Patients Health care professionals
Makai et al [38], Netherlands	Quantitative controlled before-and-after study	682 patients; frail older adults 290 patients in the intervention group 392 patients in the control group	Patients
Biese et al [46], United States	RCT^b^	120 patients who were discharged from the ED^c^; no requirements of chronic condition or diagnosis 39 patients in the intervention group 35 patients in the placebo group 46 patients in the control group	Patients Health care professionals
Mavandadi et al [47], United States	RCT	1018 patients with depression or anxiety 509 patients in the intervention group 509 patients in the control group	Patients
Gurwitz et al [48], United States	RCT	3661 patients who were discharged from the hospital (some had no diagnosis or chronic conditions, and others had diabetes, myocardial infarction, heart failure, COPD, cancer, stroke, cerebrovascular disease, or renal disease) 1870 patients in the intervention group 1791 patients in the control group	Patients
Gellis et al [39], United States	RCT	115 patients with either heart failure or COPD and screened for depression 57 patients in the treatment group 58 patients in the control group	Patients
Gellis et al [40], United States	RCT	115 patients with either heart failure or COPD and screened for depression 57 patients in the treatment group 58 patients in the control group	Patients Health care professionals
Cutrona et al [49], United States	Mixed methods; descriptive quantitative study and qualitative focus group study	799 patients who were discharged from the hospital (diagnosis not mentioned in the article) Focus group with 5 physicians	Patients Health care professionals
Makai et al [37], Netherlands	Mixed methods; quantitative study and qualitative individual interviews	290 patients; frail older adults 23 of these patients and their informal caregivers were included in semistructured individual interviews	Patients Health care professionals
Mateo-Abad et al [50], Spain	Mixed methods; quantitative and qualitative study	200 patients with two or more chronic conditions (at least one of them being COPD, chronic heart failure, or diabetes mellitus) 101 patients in the intervention group 99 patients in the control group 9 qualitative interviews with patients, carers, clinicians, nurses, and managers	Patients Health care professionals
Dent and Tutt [51], United Kingdom	Longitudinal qualitative study	44 IT and health care professionals’ experiences with implementation and integration of an IT-supported care pathway for frail older adults	Health care professionals
Freilich et al [52], Sweden	Qualitative study	42 participants 12 patients with multimorbidity (one chronic condition was either heart failure or diabetes mellitus) 3 registered nurses 20 physicians 7 family caregivers	Patients Health care professionals

^a^COPD: chronic obstructive pulmonary disease.

^b^RCT: randomized controlled trial.

^c^ED: emergency department.

In total, 44% (7/16) of the studies were conducted in the United States [39,40,42,43,46-49]. A total of 12% (2/16) of the studies were conducted in the Netherlands [37,38,45], and 12% (2/16) were conducted in the United Kingdom [41,51]. In total, 6% (1/16) of the studies were conducted in each of the following three countries: Ireland [44], Spain [50], and Sweden [52]. Of the 16 articles, 7 (44%) were published in 2014 [35,37-39,41,45,50], and 4 (25%) were published in 2017 [44,49] and 2018 [41,45]. However, no studies were included from 2019 or 2021. A total of 12% (2/16) of the studies were published in 2020 [50,52].

Most studies (12/16, 75%) had a quantitative design [38-48] (Table 1). Among the 16 studies, there were 5 (31%) quantitative pilot or observational studies [41-45], 5 (31%) RCTs [39,40,46-48], and 1 (6%) before-and-after study [38]. A total of 19% (3/16) of the studies were mixed methods and combined qualitative and quantitative data [37,49,50], and 12% (2/16) had a qualitative design [51,52].

The included patients had a variety of chronic illnesses or multimorbidity, such as heart failure, chronic obstructive pulmonary disease, diabetes, cancer, dementia [39,40,44,45,48,50,52], frailty [37,38,41,44,51], or depression or anxiety [39,42,47]. A total of 56% (9/16) of the studies had participants who were older adults [37-40,43,44,46-48]. In total, 25% (4/16) of the studies were limited to older adults. However, this 25% (4/16) of studies also collected data on health care professionals’ use of the eHealth intervention (how many times health care professionals opened alerts or accessed an electronic health portal) [37,40,45,46]. A total of 31% (5/16) of the studies included both older adults and health care professionals such as nurses, general practitioners, and health managers [41,42,49,50,52]. In total, 6% (1/16) of the studies were limited to health care professionals and focused on electronic integrated e-pathways for frail older adult patients [51].

### Categories of eHealth and CC Activities

#### Overview

We identified three categories of eHealth in CC for older adults living at home in the included studies: (1) EHRs and patient portals, (2) telehealth monitoring solutions, and (3) telephone only. In the EHRs and patient portals category, electronic journals, personal health portals, and electronic personal health plans were used in CC [37,38,43,45,48-50]. In the telehealth monitoring solutions category, virtual ward or sensor technology was used [39-42,44,51,52]. In all these studies (7/7, 100%), sensor technology and telehealth monitoring were combined with electronic portals or home visits [39-42,44,51,52]. Telephones only were used in 12% (2/16) of the studies [46,47]. See Multimedia Appendix 2 [37-52] for an overview of the eHealth interventions or solutions used in the studies and the identified CC activities.

#### EHRs and Patient Portals

A total of 44% (7/16) of the studies [37,38,43,45,48-50] were classified in the EHRs and patient portals category. Logue and Effken [43] described barriers and facilitators when older adults used a personal health record to manage their health. De Jong et al [45] evaluated health care professionals’ use of an electronic health portal (Congrendi). Makai et al [37,38] conducted an intervention on a health and welfare information portal (ZWIP) for frail older adults. Cutrona et al [49] explored the use of electronic messages sent upon hospital discharge of older adults and received by primary care physicians in the EHR. Mateo-Abad et al [50] conducted and evaluated an intervention including an electronic personal health folder with information, education, care plans, electronic messages between older adults (or their carers) and health care professionals, and monthly telephone and face-to-face meetings. Gurwitz et al [48] measured the effect of using EHRs and sent automatic alerts to the primary care health care professionals when older adults were discharged from hospital to home.

In Table 2, CC activities according to the Care Coordination Atlas are described. Communication and information exchange is evident in 100% (7/7) of the studies. The study by Mateo-Abad et al [50] included several CC activities, for example, support for self-management goals where the patients were educated and guided on managing their chronic illness by using the health portal and over the telephone. Furthermore, the patients reported personal health data in the health portal [50]. In many studies (6/7, 86%), health care professionals received automatic alerts about information such as new medication, test results, or recommendations on treatment registered in the EHRs and patient portals [37,38,43,45,48,49]. Automatic alerts were related to the CC activities communication and information exchange, facilitation of transitions, monitoring, following up, and responding to change.

**Table 2:** Overview of Care Coordination Atlas activities in the electronic health records (EHRs) and patient portals category.

Care Coordination Atlas activities	EHRs and patient portals
Establish accountability or negotiate responsibility	Patients were responsible for who they wanted to add to their electronic health portal [37,38]. Patients had to give permission to begin a record and invite health care professionals to sign up. However, patients themselves did not use the eHealth portal [45].
Communicate	Health care professionals and patients, or professionals and other health care professionals communicated with the help of electronic messages [45,49,50]. Contact with the patient through telephone or in person [37,38,45,50] Health care professionals received automatic digital alerts about relevant patient health information [37,38,43,45,48,50].
Facilitate transitions	Primary care health care professionals received automated alerts when a patient was discharged from the hospital regarding discharge information, new drugs, medication warnings, and notification to schedule a follow-up appointment [48,49].
Assess needs and goals	Patients could register care-related goals in the electronic care plan and initiate a change in the plan when a goal was reached [37,38].
Create a proactive plan of care	By using and accessing an electronic personal health plan, patients were more involved and responsible for their health [50].
Monitor, follow up, and respond to change	Health care professionals followed up on clinical information that was registered in the health portal [50].
Support self-management goals	Patients received education and guidance on managing their chronic illness over the telephone or in the health portal [50].
Link to community resources	Not evident
Align resources with patient and population needs	Health care professionals in primary care experienced an increased workload with the new eHealth model [50].

#### Telehealth Monitoring Solutions

The category of telehealth monitoring solutions, such as sensor technology and virtual wards, was evident in 44% (7/16) of the studies [39-42,44,51,52]. Gokalp et al [41] focused on piloting a telemonitoring system for older adults, including various sensors such as pulse sensors, bed sensors, glucose meters, and blood pressure (BP) meters. Sheeran et al [42] tested the feasibility, acceptability, and clinical outcomes of a telemonitoring technology for older adults with depression. Lewis et al [44] monitored and tested a community ward integrating specialist and primary health care. Gellis et al [39,40] evaluated and examined the impact of a telehealth monitoring intervention, including a tabletop monitor at the homes of older adults where they could register weight, BP, pulse, and other vital signs. In addition, a health care professional conducted depression treatment sessions over the telephone for older adults with comorbid depression [40]. Dent and Tutt [51] reported health care professionals’ experiences with an e-care pathway, including a virtual ward and telemonitoring of a patient in their home. Freilich et al [52] explored the perspectives of health care professionals, patients, and caregivers on the use of a telemedicine program, including telehealth monitoring (BP, weight, and blood sugar) and the use of a tablet to conduct video meetings with a nurse.

CC activities such as communicating and exchanging information, facilitating transitions, monitoring, following up, and responding to change were evident in all studies (7/7, 100%), as described in Table 3. In the studies by Lewis et al [44] and Dent and Trutt [51], the CC activity to facilitate transition was apparent as information on patient transfer was electronically sent from specialist to primary health care. Telephone and EHRs and patient portals were combined with telehealth monitoring, where health care professionals conducted education or counseling sessions over the telephone, video, or in EHRs and patient portals [39,40,42,52].

**Table 3:** Overview of Care Coordination Atlas activities in the telehealth monitoring solutions category.

Care Coordination Atlas activities	Telehealth monitoring solutions
Establish accountability or negotiate responsibility	A telehealth nurse was assigned to be a care manager for the patient and contacted other health care professionals when necessary [42]. A senior nurse was appointed as the clinical care manager and was responsible for patient care [44]. Some patients reported not knowing if primary health care professionals or hospital specialists communicated with each other [52].
Communicate	Education or counseling sessions were conducted over the telephone [39,40,42,52]. The studies used a variation of home visits, video meetings, or telephone calls to patients or health care professionals [41,42,44,51,52]. Patients’ health data were registered in a portal and reviewed by a nurse [41,52].
Facilitate transitions	Information about the patient was sent to primary health care when the patient was transferred between specialist and primary health care or needed a change in treatment [44,51]. Telehealth nurses contacted and referred patients to primary care health care professionals when they observed changes in patients’ health data [41]. Different health care professionals were located together, and a care manager followed up with the patient across specialist and primary care [44]. If a patient was discharged from hospital to home, a community nurse received an alert in an electronic portal and would ensure early discharge of the patient [51].
Assess needs and goals	A telehealth nurse provided goal setting over the telephone with patients [42].
Create a proactive plan of care	Not evident
Monitor, follow up, and respond to change	Health care professionals monitored and assessed patient health data that were registered in an eHealth portal [39-42,44,51,52]. Some patients felt secure knowing that a nurse kept track of their health parameters and would contact them if changes were observed [52].
Support self-management goals	Patients received education or counseling sessions over the telephone or via an eHealth portal [39,40,42,52]. Patients could ask questions or discuss a problem with a telehealth nurse when needed [39,40].
Link to community resources	Not evident
Align resources with patient and population needs	A virtual ward model with telehealth monitoring was set up with existing resources [44].

#### Telephone Only

A total of 12% (2/16) of the studies belonged to the third category, telephone only [46,47], and the telephone was used in combination with EHRs, patient portals, and telehealth monitoring solutions [39-42,44,50], as reported in Tables 2 and 3. As shown in Textbox 2, support for self-management goals was evident in the study by Mavandadi et al [47], where a nurse conducted symptom monitoring, education, and problem-focused therapy for older adult patients with depression and anxiety over the telephone. The study was an RCT; however, >20% of the included patients did not complete the intervention because of reduced cognitive function [47]. The RCT study by Biese et al [46] evaluated a telephone intervention in which older adults were telephoned within 5 days of an ED visit regarding making an appointment with physicians or medication changes. In this study, approximately 10% of the patients could not be reached by telephone after 3 attempts [46].

**Overview of Care Coordination Atlas activities in the telephone only category.**

**Care Coordination Atlas activities identified**

- Establish accountability or negotiate responsibility: not evident
- Communicate: health care professionals contacted patients over the telephone [46,47]
- Facilitate transitions: a nurse telephoned patients 3 days after discharge from hospitals and helped patients who needed it navigate the health care system by reviewing discharge instructions and making appointments with or referrals to physicians [46]
- Assess needs and goals: not evident
- Create a proactive plan of care: not evident
- Monitor, follow up, and respond to change: health professionals monitored response to treatment and facilitated treatment over the telephone with patients [47]
- Support self-management goals: a study nurse conducted symptom monitoring, education, and problem-focused therapy with patients over the telephone [47]
- Link to community resources: not evident
- Align resources with patient and population needs: not evident

### Patient-Level and System-Level Outcomes

Overall, 56% (9/16) of the studies measured the effect on patient- or system-level outcomes when implementing, piloting, or testing an eHealth solution [38-40,42,44,46-48,50]. See Table 4 for a detailed description of the interventions and outcomes. The patient-level outcomes were related to physical or mental health and social or problem-solving skills [38-40,42,47,50]. The system-level outcomes were related to health care use, such as hospitalizations, readmissions, follow-up visits with primary care health care professionals, or ED admission rates [39,44,46,48,50]. The patient-level outcomes were measured using standardized scales and survey questionnaires or recording vital signs throughout the intervention [38-40,42,47,50]. System-level outcomes were measured with objective scores, such as how often or if the patient went to the general practitioner or differences in hospitalization or ED visit rates between the intervention and control groups [39,44,46,48,50].

The patient- and system-level outcomes were mixed. Of the 9 studies, 7 (78%) showed improved physical or mental health [39,40,42,47,50], improved social and problem-solving skills [39,40], lower hospitalization rates, lower ED visits [39,44,46], and increased follow-up rates with primary care health care professionals [46,50]. In total, 11% (1/9) of the studies demonstrated no differences in physical or mental health between the intervention and control groups [38]. Makai et al [38] included 682 older adults in their study with a control and intervention group. The study by Gurwitz et al [48] did not demonstrate an increase in follow-up visits with primary care health care professionals or a reduction in rehospitalization rates. This study included >3661 patients but did not explore ways of communicating directly with patients or health care professionals other than sending automatic alerts to health care professionals [48].

**Table 4:** Overview of interventions and identified patient-level and system-level outcomes.

Intervention description	Patient-level outcomes	System-level outcomes
Mateo-Abad et al [50] conducted and evaluated the effect of an electronic personal health folder, which included accessing information, electronic messages, web-based education, monthly telephone calls, and face-to-face sessions with nurses. The intervention group used the electronic personal health folder and received usual care. The control group only received usual care. Outcomes related to clinical effect and the use of services were measured at two points throughout the intervention period (9 and 12 months). EHR^a^ and administrative databases were used to extract available information.	Health data levels (BMI, blood pressure, blood glucose, and oxygen saturation) were significantly reduced in the intervention group compared with the control group [50].	There were lower hospitalization rate and increased appointments with general practitioners and nurses in the intervention group compared with the control group [50].
Makai et al [38] conducted a controlled before-and-after study of the health and welfare portal ZWIP. ZWIP contains a secure electronic messaging system and an EHR where the patient can invite health care professionals and their caregiver to join. Data were collected using a questionnaire with patients and their families at baseline and after 12 months. The control group received usual care.	The researchers observed no differences in physical or mental health between the intervention and control groups [38].	N/A^b^
The study by Gurwitz et al [48] assessed the effect of an EHR intervention. In the intervention group, automatic alerts were sent to the primary care health care professionals when older adults were discharged from the hospital. Data were collected on whether discharged individuals had an office visit with a primary care physician in the 7-, 14-, and 30-day periods after hospital discharge. The primary care health care professionals did not receive automatic alerts or information when older adults in the control group were discharged.	N/A	The study did not demonstrate an increase in follow-up visits with primary care health care professionals or a reduction in rehospitalization [48].
The quantitative pilot study by Sheeran et al [42] consisted of testing the feasibility, acceptability, and preliminary clinical outcomes of a telemonitoring technology to provide depression care. Data from older adults were collected at baseline and at the discharge of the intervention, which lasted a minimum of 3 weeks. Older adults had telephone contact or home visits by a telehealth nurse.	19 older adults had severe depression after the intervention, and 16 of them reported a mild depression score after the intervention [42].	N/A
Gellis et al [39] conducted an RCT^c^ that tested the intervention, including the Honeywell Health Monitoring system. Weight, blood pressure, pulse, oxygen saturation, and temperature were monitored daily. A telehealth nurse was available for the older adults daily and monitored the data. Information from the older adults was collected using study questionnaires at baseline and at approximately 3 months. The control group received usual care.	Patients in the intervention group showed a greater increase in general health and social functioning than patients in the control group after 3 months [39].	The control group in their RCT study had significantly more visits to the ED^d^ than the intervention group after 3 months [39].
Gellis et al [40] conducted an RCT that tested the intervention, including the Honeywell Health Monitoring system. In addition, the intervention group received chronic illness and depression care management and problem-solving treatment. A telehealth nurse monitored data and completed problem-solving treatment over the telephone with the older adults. A satisfaction survey, depression rating scale, and other information were collected at baseline and 3 and 6 months.	Results showed that the intervention group had greater problem-solving abilities, and their depression symptom scores improved significantly compared with those of the control group at the 3-month survey [40].	N/A
Lewis et al [44] conducted a quantitative observational study of a virtual ward using telehealth monitoring solutions. The virtual ward monitored older adults with home visits and telephone consultations. The risk of hospital admission was measured upon admission to the virtual ward. The number of unplanned admissions and ED presentations was measured before starting the intervention and upon discharge from the virtual ward.	N/A	The study demonstrated a reduction in ED visits and unplanned hospital admissions [44].
Mavandadi et al [47] conducted an RCT where the older adults in the intervention group received telephone-delivered symptom monitoring and were provided with educational and problem-focused therapy. The intervention group received maintenance calls at the 4-, 5-, and 6-month follow-ups. Both the control and the intervention group received 4 brief follow-up assessments over the telephone.	The older adults in the intervention group reported greater improvement in overall mental health functioning and reduced anxiety and depressive symptoms compared with those in the control group [47].	N/A
Biese et al [46] conducted an RCT that evaluated a telephone call intervention conducted by a trained nurse 1 to 3 days after ED discharge. The nurse followed a script and helped patients review discharge instructions and arranged appointments with physicians when needed. The placebo group received a satisfaction survey call 1 to 3 days after ED discharge, and the control group received no call. Telephone interviews were conducted with all groups 5 to 6 days and 30 to 35 days after ED discharge.	N/A	The older adults in the intervention group were more likely to see a physician within 5 days compared with the control and placebo groups [46]. The study further showed a reduction in the number of registered admissions in an ED; however, this was not significant compared with the control and placebo groups [46].

^a^EHR: electronic health record.

^b^N/A: not applicable.

^c^RCT: randomized controlled trial.

^d^ED: emergency department.

### Facilitators of and Barriers to the Use of eHealth in CC

In the analysis of the articles, we identified two factors—facilitators and barriers—describing the use of eHealth in CC. A total of 8 facilitators and barriers were identified (see the overview in Textbox 3). Some of these barriers and facilitators were reported from patient satisfaction surveys [39,40,42]; descriptions of how health care professionals or older adults used the eHealth solution [46,47,50,51]; or qualitative data on experiences, evaluation, and use [37,41,49,50,52].

**Overview of facilitators of and barriers to the use of eHealth in care coordination.**

**Facilitators**

- Available support to the patient
- Relation continuity between the older adult and health care professional
- A sense of security and safety
- New and valuable way to observe changes in patients’ health

**Barriers**

- Individual characteristics
- Lack of experience, knowledge, or confidence regarding how to use eHealth
- Increased workload
- Hampered communication because of limited access to the electronic health records or patient portals

Available support to the patient was an important facilitator for older adults’ use and management of eHealth technology [37,46,52]. Biese et al [46] reported that some older adults needed assistance to book appointments with health care professionals over the telephone. Makai et al [37] reported that some older adults had problems logging in to the electronic health portal, pointing out the importance of having available support to the patient. Freilich et al [52] claimed that some patients needed health care professionals to be in control and monitor their symptoms. Other patients did more of the monitoring and disease management themselves, making it necessary for health care professionals to tailor their support to the patients [52].

Relational continuity between the older adult and health care professional was important to facilitate the older adult’s use of eHealth. In total, 12% (2/16) of the studies [50,52] reported that a close relationship between health care professionals and older adults supported the development and follow-up of electronic care plans. Another aspect highlighting the importance of relational continuity was that some older adults feared that eHealth would replace face-to-face contact, potentially negatively affecting eHealth use [35,52].

The use of eHealth in CC among older adults was also facilitated when they felt a sense of security and safety. A total of 19% (3/16) of the studies [37,38,50] reported that older adults felt reassured knowing that health care professionals were keeping track of their health data. In addition, being in charge of their health symptoms and communicating directly with health care professionals gave older adults a sense of safety and security [37,38,50].

Health care professionals’ use of eHealth to coordinate care was facilitated when it was experienced as a new and valuable way of observing changes in patients’ health [40,41,50]. In the study by Gellis et al [40], the nurses were attentive to changes in patient health by reviewing the health portal daily. Mateo-Abad et al [50] reported that the nurses who monitored the patient health data had greater familiarity with the older adults’ chronic illnesses.

Individual characteristics such as being an older adult and having health problems such as hearing impairment and memory loss were barriers to the use of eHealth [38,43,47,50,52]. Adults aged ≥80 years did not use technology as often as younger older adults [38,43,47,51,52]. Mavandadi et al [47] reported that 23.6% of the 1018 included patients did not pick up the phone despite several attempts and having been given information about the study beforehand. According to Makai et al [38], most of the included patients rarely used the eHealth solution for coordination despite efforts from the researchers to implement and train them in its use.

The use of eHealth by older adults was also limited by a lack of experience, confidence, and knowledge of how to use it [41,43,52]. The patient group aged >80 years can be perceived as heterogenic and less confident in using technology than younger older adult patients [43]. Furthermore, Logue and Effken [43] found that more men than women expressed confidence in their ability to use technology.

Increased workloads limited health care professionals’ use of eHealth. Some health care professionals experienced a heavier workload when implementing a new eHealth tool such as a patient portal [50]. In the studies by Biese et al [46] and Dent and Tutt [51], a dedicated nurse was in charge of facilitating transitions from hospital to home. The nurse arranged appointments and referrals and reviewed discharge instructions with the care team and the patient. Cutrona et al [49] found that primary care physicians perceived alerts in the EHR inbox as burdensome and, if the physicians had too many alerts in their inbox, the alert was less likely to be opened within 24 hours.

eHealth used for CC communication was hampered by limited access to the EHRs and patient portals for the health care professionals. De Jong et al [45] reported that less than half of the included patients had general practitioners linked to the EHR. The EHR was an additional system to what the general practitioners already used. A similar finding was reported in the studies by Makai et al [37,38], where patients could register care-related goals in a web-based care plan. In this study, not all health care professionals signed up to the portal or answered messages from the patients. In the study by Freilich et al [52], patient and family caregivers entered personal health data into the telehealth monitoring solution. However, this information was sent only to the primary health center. The primary health center belonged to the health region, but the home care nurses who visited the patients were employed by the municipality and did not have access to this information [52].

## Discussion

### Principal Findings

This review mapped the research literature on eHealth in CC for older adults living at home. We included 16 articles in the scoping review and identified three categories of eHealth: EHRs and patient portals, telehealth monitoring solutions, and telephone only. Communication was the CC activity reported in all the articles (16/16, 100%). Patient- and system-level outcomes were mixed. Most studies (7/16, 44%) showed that improved mental and physical health, reduced rehospitalization and hospital admissions, available support to the patient, relational continuity with health care professionals, and a sense of security were facilitators of older adults’ use of eHealth in CC. Having new and useful tools for observing a change in patients’ health facilitated health care professionals’ use of eHealth in CC. Individual characteristics and lack of experience, confidence, and knowledge were barriers to older adults’ use of eHealth in CC. Barriers reported by health care professionals were increased workload and hampered communication because of limited access to the EHRs and patient portals.

### Comparison With Prior Work

We identified 3 eHealth categories when coordinating care for older adults. Despite the fast development of eHealth and technology, our results indicate that the telephone should still be considered necessary for older adults. A total of 12% (2/16) of the articles were classified under the category of telephone only [46,47]. However, the telephone was used in combination with EHRs, patient portals, and telehealth monitoring in 38% (6/16) of the studies [39-42,44,50]. Hawley et al [53] reported that, for older adults who were uninterested in and incapable of using eHealth, the telephone was important in the conduct of digital home visits. This is also supported by the study by Chu et al [54], where almost one-fifth of the older adults in the study did not have access to an electronic device, leaving the telephone as the only option to conduct virtual visits. EHRs and patient portals are commonly used in CC and integrated care programs, which is supported by other studies [11,55]. Melchiorre et al [55] categorize monitoring as an eHealth solution in integrated care programs, which supports the identification of the category of telehealth monitoring solutions.

Our results showed that communication was the dominant CC activity in all 3 eHealth types, a finding supported by other studies using the Care Coordination Atlas as a framework [56-58]. Both health care professionals and older adults communicated through electronic messages, over the telephone, via video, or in person. McDonald et al [11] highlighted that EHRs ensure information transfer between health care professionals. Our results document that facilitating transitions, supporting self-management goals, monitoring, following up, and responding to change are common CC activities in the 3 eHealth categories. Similarly, Chakurian and Popejoy [58] found the same CC activities when they used the CC framework to evaluate transitional care models. However, our review did not identify the CC activity community resources in the included eHealth types. This contrasts with the study by Samal et al [59], which reported the CC activity community resources as helpful regarding the automatic reference of patients to community programs when discharged from the ED or hospital. The Chronic Care Model also highlights that better access to community resources is important for individuals with chronic conditions [60].

A CC activity that appeared often was establishing accountability or negotiating responsibility [37,38,42,44,45,52]. Findings from our review show that older adults consented to who could access or start an electronic record in 19% (3/16) of the studies [37,38,45]. According to Tith et al [61], health care services still have challenges with patient consent. In Europe and the United States, giving consent and overseeing who can access personal health information are included in policy standards such as the Health Insurance Portability and Accountability Act [53] and the General Data Protection Regulation [62]. However, Samal et al [59] stated that the activity of establishing accountability or negotiating responsibility has a low future potential to be used in HIT as it cannot be automated. However, the results of this scoping review show that, when using eHealth to coordinate care, it is essential to ensure that patients know what information is shared about them and with whom.

In total, 56% (9/16) of the studies measured the effect of the eHealth interventions. Some of the studies focusing on patient outcomes (5/6, 83%) showed greater social functioning and improved mental and physical health [39,40,42,47,50], indicating a greater quality of life [11]. McDonald et al [11] described that the end point of CC measures is, among other things, improved quality of life and reduced hospital readmissions and emergency room visits, which is in line with the patient and health care use outcomes of this scoping review.

We identified that older adults’ characteristics, such as being very old and having health problems and memory loss, were barriers to eHealth use. Anderson and Perrin [63] reported that, even though more older adults than ever use smartphones in the United States, seniors aged 65 to 69 years are more likely to go on the web than those aged ≥80 years. Our results indicate that these older adults did not use technology as often as younger older adults, which can be described as the *digital divide* [63-65]. In addition to being older and less educated, impaired cognitive and numeracy ability, limited internet experience, and physical and visual impairment can limit the use of eHealth [66,67].

The digital divide can be explained in relation to eHealth literacy [68], where Rios et al [69] emphasized that training older adults in the use of technology can increase eHealth literacy. A recent mixed methods study by Fox and Connolly [70] found that it is important to educate older adults about mobile apps, wearable devices, and EHRs. Our results showed that available support to the patient and relational continuity could facilitate older adults’ use of eHealth. Kim and Lee [71] reported limited information about training and support for patients when using electronic devices, which can hamper eHealth use [69]. Vroman et al [72] and Hawley et al [53] emphasized that training and education need to be personalized to the older adult’s needs and skills. Sufficient technological support is also important to increase health literacy and narrow the digital divide when using eHealth [70,73].

A barrier that health care professionals reported was increased workloads and having new work tasks assigned related to the use of eHealth in CC. Gill et al [74] reported that health care professionals made significant efforts to gather patient information when using HIT to facilitate CC. According to Greenhalgh et al [75], new technologies can disrupt work processes, and some health care organizations cannot adapt to new ways of working.

The lack of interoperability across health systems hampers information exchange and communication when using eHealth in CC [9,11,74,76]. Hsiao et al [77] reported that office-based physicians who used HIT did not always receive the necessary patient information to coordinate care, especially from health care professionals outside their practice or hospital. Moreover, Liaw et al [76] addressed that the lack of a universal secure messaging system causes fragmented information sharing among health care professionals [9,59,77].

### Future Directions

Our results showed that CC activities, including identifying community resources, establishing accountability, and negotiating responsibility, have a future potential for inclusion in eHealth research and practices. We recommend that community resources such as volunteer work, food delivery services, and support groups [11] be considered in both future research and practice. Furthermore, the digitalization of consent and responsibility can be enabled using a digital e-consent solution where patients can create, update, or withdraw their consent [61].

To narrow the digital divide and take into account the variety of older adults’ individual characteristics, future researchers, practices, and policy makers need to consider using the telephone or in-person visits as a supplement or backup when conducting virtual visits or telehealth monitoring in CC. To meet the individual needs of older adults, customized support to the patient and education can be helpful to the successful use of eHealth in CC. Knowledge, confidence, and support are needed to ensure patient involvement when using eHealth to coordinate care. Therefore, future practice should have health care professionals with dedicated responsibility and time to individually follow up on older adults. This can ensure sufficient allocation of resources in CC when using and introducing eHealth. As previously mentioned, interoperability is still an issue. Policy makers and practices should continuously focus on ensuring access for all health care professionals to common CC eHealth solutions such as EHRs or patient portals.

### Strengths and Limitations

In this scoping review, several limitations need to be addressed. First, we did not critically appraise the included studies as scoping reviews are flexible in their methodology [32] and are not always conducted when the aim is to map evidence [78]. By not critically appraising the studies, we included studies that varied greatly in the number of participants and methodological approaches. Therefore, our results should not be generalized. The results can be seen as important for future research and practice, policy makers, and the development of new eHealth tools to coordinate care for older adults. Furthermore, scoping reviews can include gray literature such as policies or government documents [32]. We excluded gray literature from this review as we aimed to map the research literature given the strong policy push to use eHealth [79].

Second, we searched 4 databases and used several search terms relevant to CC, eHealth, home care, and older adults. Our searches were conducted with the assistance of an experienced librarian. Despite our efforts to map the research literature on eHealth and CC for older adults living at home, we may have missed some studies.

Third, we included studies with participants aged ≥65 years and excluded several studies because the participants were younger (eg, aged 60 years). The World Health Organization [3] is moving away from using a chronological definition of old age (eg, 65 years). However, our decision was based on the need for knowledge on the use of eHealth in CC among older adults living at home [30].

### Conclusions

The number of older adults will continue to increase well into the future. Older adults with chronic illnesses must navigate fragmented health care services, and eHealth in CC may be a way to prevent this fragmentation. The use of eHealth in CC for older adults is promising, although the outcomes so far have been mixed. eHealth in CC may improve older adults’ mental and physical health and reduce hospital admissions and readmissions. A barrier was hampered communication because of the lack of interoperability of the EHRs and patient portals, which seems to be an ongoing issue worldwide.

To ensure the successful use of eHealth in CC for older adults living at home, the eHealth used needs to be customized to each individual’s care needs. Education and patient support should be individualized. The telephone is still important for some older adults, and future research and practice should consider using the telephone or in-person visits to close the digital divide. However, it is essential to ensure that older adults interested in and capable of using HIT can be offered eHealth in CC. This calls for individualized eHealth-enabled health care services for older adults. eHealth in CC has an immense potential for the future organization and development of health care services. Thus, more in-depth knowledge of eHealth at the crossroads of CC for older adults living at home is needed.

## Appendix

Multimedia Appendix 1 Example of a search.

Multimedia Appendix 2 Overview of the included papers and care coordination activities.

## Abbreviations

BP: blood pressure
CC: care coordination
ED: emergency department
EHR: electronic health record
HIT: health IT
PRISMA: Preferred Reporting Items for Systematic Reviews and Meta-Analyses
PRISMA-ScR: Preferred Reporting Items for Systematic Reviews and Meta-Analyses extension for Scoping Reviews
RCT: randomized controlled trial
