# Supplementary material for: eHealth in Care Coordination for Older Adults Living at Home: Scoping Review
Source: J Med Internet Res. 2022 Oct 18;24(10):e39584. doi: 10.2196/39584 (PMC9627466; doi:10.2196/39584)
Supplement: Multimedia Appendix 1 [file jmir_v24i10e39584_app1.docx]

**Appendix A – example of a search**

|  | **text words** |
| --- | --- |
| **SEARCH #1** |  |
|  | "care coordination" |
|  | "coordinated care" |
|  | "integrated care" |
|  | "integrated health" |
|  | "care management" |
|  | "patient care management" |
|  | "case management" |
|  | "care process" |
|  | "collaborative care" |
|  | "care pathway*" |
|  | "care tranistion*" |
|  | "continuity of care" |
|  | "care planning" |
|  | "continuum of care" |
|  | "shared care" |
|  | **search 1 #combine with OR** |
| **SEARCH #2** |  |
|  | "ehealth or e-health" |
|  | "telecare" |
|  | "telehealth" |
|  | "telemedicine" |
|  | "remote consultation*" |
|  | "assistive technolog*" |
|  | "electronic health record*" |
|  | "Information communication technology" or ict |
|  | "mhealth" or "m-health" |
|  | **SEARCH 2 combine with OR** |
|  |  |
| **SEARCH #3** |  |
|  | "Homecare or home care" |
|  | "Home care servic*" |
|  | "home health care" |
|  | "Home nursing" |
|  | "community dwelling" |
|  | "independent living" |
|  | "home based care" |
|  | "community health servic*" |
|  | "municipal health servic*" |
|  | "primary health care" |
|  | "General practitioner*" |
|  | **#SEARCH 3 combine with OR** |
|  |  |
| **SEARCH #4** |  |
|  | "older patient*" |
|  | "elderly" |
|  | "aged" |
|  | "older person*" |
|  | "elderly" AND "multimorbid*" |
|  | elderly AND "chronic ilnness*" |
|  | "Older adult*" |
|  | "frail elderly" |
|  | **#SEARCH 4 combine with OR** |
|  |  |
|  |  |
|  | **Combine Search #1, #2, #3, #4 with AND** |

If possible, select following limitations in the database:

| *limit to peer review |
| --- |
| *English language |
| *2009-2020 |
