# Supplementary material for: eHealth in Care Coordination for Older Adults Living at Home: Scoping Review
Source: J Med Internet Res. 2022 Oct 18;24(10):e39584. doi: 10.2196/39584 (PMC9627466; doi:10.2196/39584)
Supplement: Multimedia Appendix 2 [file jmir_v24i10e39584_app2.docx]

Appendix B – Description of research aim, data collection, and description of e-health solution/intervention and care coordination domains

| Authors | Aims | Data collection and measurements | Description of e-health solution/ intervention | Evident Care Coordination Domains |
| --- | --- | --- | --- | --- |
| Sheeran et al [42] 2011 | To test the feasibility, acceptability, and preliminary clinical outcomes of a protocol to use home telemonitoring technology to provide geriatric DCM (depression care management) to participating elderly homecare patients | Baseline interviews at the start of interventions and follow-up interviews upon discharge | A depression telecare protocol was adapted to an already existing homecare telehealth monitor. Patients were asked about medication, and side effects in the e-health solution, and educated on what to do. A telehealth nurse had interviews and goal setting via telephone, and phone or fax-communication with PCH or other professionals. | **Establish accountability or negotiate responsibility *Communicate*  **Support self-  management goals *Assess needs and   goals* |
| Logue and Effken [43] 2012 | The purpose of the study was to describe and explore the personal barriers and facilitators of PHR adoption in this sample as a foundation for future research and application development. | Survey questionnaire and descriptive statistic analysis | A personal health record (PHR) which were adopted by older adults to self-manage their chronic conditions. Owned by patients. Information about conditions, illness, medications, and summary of health from healthcare providers. | **Communicate* |
| Biese et al [46] 2014 | To evaluate whether a telephone call intervention conducted by a trained nursed 1 to 3 days after an older adult's index ED visit would achieve enhanced care plan compliance as measured by expedited outpatient follow-up within 5 days of the index ED visit and/or increased compliance with medication changes | Telephone interviews 5-6 days post ED discharge and 30-35 days post ED discharge in all study groups.  - In the intervention group: a   call 1-3 days post ED discharge.  - In the placebo group: patient   satisfaction survey call 1-3 days   post ED. -In the control group: no call | The intervention was a telephone call 1-3 days post ED discharge from a study nurse, who followed a script and helped patients review discharge instructions and arrange appointments with doctors or services if needed. | **Communicate  *Facilitate transitions* |
| Lewis et al [44] 2017 | The study aimed to determine if unplanned hospital admission and ED presentations could be reduced through a CVW model (Community virtual ward) | The risk of hospital admission was measured on admission to the CVW. Baseline cognition and demographics. Number of unplanned admissions and ED presentations were measured prior to CVW admission and on discharge. Statistical analysis. | The CVW model was set up to work within existing resources. Patients were monitored with both home visits and telephone consultations. The CVW operated with a traffic light system, red, amber, and green. | **Establish accountability or negotiate responsibility *Communicate*  **Facilitate transitions *Monitor, follow up   and respond to   change*  **Align resources with patient and population needs* |
| Makai et al [38] 2014 | To investigate the effectiveness of an online health community (OHC) intervention for older people with frailty aimed at facilitating multidisciplinary communication. | Controlled before-after study with 12 months follow-up. A face-to-face questionnaire with family practitioner and patients at their home at the baseline and at follow-up. | ZWIP is a personal online health community for multidisciplinary communication and information exchange for frail older people and their informal caregivers. It has a secure messaging system, providers can join their network. Care-related goals and action plans can be registered in ZWIP. | **Establish accountability or negotiate responsibility *Communicate *Assess needs and   goals* |
| Makai et al [37] 2014 | To evaluate differences in the use of personal online health community (POHC) for frail older people in relation to personal characteristics, and to explore barriers and facilitators for use as experienced by older people and their informal caregivers, using the case of Health and Welfare Information Portal (ZWIP) | A face-to-face questionnaire with patients and family practitioners. Data about usage from ZWIP application over 2 years. Individual semistructured interviews. | ZWIP is a personal online health community for multidisciplinary communication and information exchange for frail older people and their informal caregivers. It has a secure messaging system, providers can join their network. Care-related goals and action plans can be registered in ZWIP. | **Establish accountability or   negotiate responsibility *Communicate *Assess needs and   goals* |
| Mavandi et al [47] 2015 | To evaluate program feasibility and MH (mental health) outcomes among community-dwelling older adults | A baseline assessment in both groups.  -The Monitor Alone (MA)   group: 4 brief follow-up   assessments  -The Care   Management (CM) group   had the same as MA. Plus care   management delivered   by behavioral health   providers over   telephone. Maintenance   calls at 4, 5 and 6-month   follow-up. | The intervention in the CM group included telephone-delivered symptom monitoring and providing educational and problem-focus therapy. | **Communicate  *Monitor, follow up   and respond to   change *Support self-  management goals* |
| Gokalp et al [41] 2018 | To investigate the potential of an integrated care system that acquires vital clinical signs and habits data to support independent living for elderly people with chronic disease. | Patient data from the sensor. Patient data have been analyzed retrospectively following hospital admission, emergency room visits, or death, to determine whether the data could predict the event. | Telemonitoring system sensors: pulse oximeter, PIR motion sensor, bed sensor, glucose meter, weight scale, medication dispenser, BP meter. Home gateway with connection to a remote server. The remote server and the clinical portal were used by the clinical team to manage patient health data. Visual alert for high BP, low Sp02, or significant change in weight in the clinical portal. The portal was reviewed daily by a nurse. | **Communicate  *Monitor, follow up   and respond to   change *Facilitate transitions* |
| Gurwitz et al [48] 2014 | To assess the effect of an electronic health record-based transitional care intervention involving automated alerts to primary care providers and staff when older adults were discharged from the hospital | Discharged individuals’ characteristics were collected, comorbidity scores, and types of diagnosis. Outcome measures related to whether discharged individuals had an office visit with a primary care physician in the 7-, 14-, and 30-day periods after hospital discharge were determined. | EHR intervention was an automated system developed to facilitate the flow of information (new drugs, recommendation of dose changes, alerts to schedule a posthospitalization office visit within 1 week). Messages were delivered on Day 3 after discharge from the hospital. | **Communicate *Facilitate transitions* |
| Gellis et al [39] 2012 | To examine the impact of a multifaceted telehealth intervention on health, mental health, and service utilization outcomes among homebound medically ill older adults diagnosed with HF or COPD. | Study questionnaires at baseline and approximately 3 months. | Intervention: Honeywell Health Monitoring System, a small tabletop in-home monitor, and a central station located at the home health care agency. Daily monitoring of weight, blood pressure, pulse, oxygen saturation, and temperature. Education and counseling about the importance of daily monitoring. A telehealth nurse was available to the patient daily, by telephone, or during urgent home visits. The telehealth nurse monitored the data and contacted the patient if there were abnormal findings. | **Communicate *Monitor, follow up   and respond to   change*  **Support self-  management goals* |
| Gellis et al [40] 2014 | To evaluate an integrated telehealth intervention to improve chronic illness and comorbid depression in the home healthcare setting. | Satisfaction survey, Hamilton depression rating scale, and PHQ-9, medical outcomes study 12item short-form survey, 12 physical and mental component subscales (PCS, MCS), social problem-solving inventory- reviser at baseline, 3 and 6 months, and a health utilization review was conducted at 12 months. | Honeywell Health Monitoring System, a small tabletop in-home monitor, and a central station located at the home health care agency. Daily monitoring of weight, blood pressure, pulse, oxygen saturation, and temperature. Chronic illness and depression care management and problem-solving treatment (PST) for comorbid depression. A nurse monitored symptoms and completed a telehealth PST session over the telephone | **Communicate *Monitor, follow up   and respond to   change*  **Support self-  management goals* |
| Dent and Tutt [51] 2014 | The article report on how health professionals and information specialists within two National Health Services (NHS) primary care trusts (PCT) implemented and utilized information technology (IT) and IT-supported care pathways (e-care pathways). | Interviews, observation meetings with a focus on the development of electronic integrated care pathways (e-ICP), and documents. | In the e-care pathway, an elderly person who had would undergo a rapid assessment to identify whether he or she can be treated in the virtual ward by being monitored via telehealth by a nurse and 24-hour care for the first couple of days if needed. When a patient had recovered sufficiently, they would be virtually discharged, however not have to leave their home. | **Communicate *Monitor, follow up   and respond to   change *Facilitate transitions* |
| de Jong et al [45] 2018 | To evaluate whether providers used the tool adequately, by measuring whether the use differs between complex and less complex care situations. | Data was collected from the Congredi systems over 42 weeks. Relevant data collected were demographics, diagnosis (dementia), numbers of providers, type of activities, and frequency of activities. | Congredi includes an interactive multidisciplinary care plan and a secure e-mailing channel. A patient had to give permission to begin a record and to invite providers to sign up. A provider opened a record and filled in a care plan, however, the patients did not directly use the solution. Providers received alerts when there were e-mails in their inboxes. | **Establish accountability or   negotiate responsibility*  **Communicate* |
| Cutrona et al [49] 2017 | To characterize factors associated with the opening of non-interruptive time-sensitive alerts delivered into primary care provider (PCP) InBaskets | A secondary analysis of primary care provider opening of time-sensitive study-generated alerts for older patients following hospital discharge. | Time-sensitive alerts were sent to physicians' secure InBaskets in the electronic health record 3 days following hospital discharge. An interface engine was linked to the hospital’s admission, discharge, transfer, and registration system. Alerts were generated for patients who had (a) new drugs added upon hospital discharge, and (b) a medication concern identified. | **Communicate*  **Facilitate transitions* |
| Freilich et al [52] 2020 | The aim of the study was to explore professionals', patients', and family caregivers' perspectives on how PHC professionals should support self-management in patients with multimorbidity. This study also includes experiences of using telemedicine to support self-management. | Interviews with a focus group of PHC professionals, pari interviews with patients and family caregivers, and individual interviews with registered nurses, family caregivers, and patients | A telemedicine program with registrations of health parameters in a tablet computer (blood pressure, weight, temperature, oxygen saturation, and blood sugar). Patients filled out questions about their health condition and symptoms. Two RNs monitored patients' registrations daily. If observed changed, they could contact patients or involve their PHC physician or home care nurse. Patients could use the tablet for video meetings with the RN. | **Establish accountability or   negotiate responsibility*  **Communicate *Monitor, follow up   and respond to   change*  **Support self-  management goals* |
| Mateo-Abad et al [50] 2020 | The aim of the study was to evaluate the impact of the CareWell integrated care model for older patients with multimorbidity | Patients were followed up for a period between 9 to 12 months. The EHR and administrative databases were used to extract available information on clinical outcomes at two-time points, and on the use of services. The qualitative evaluation took place after the intervention period, with semi-structured interviews | The intervention included usual care (consultation with GP, home visits, drug prescription, referral, patient education, and electronic health records and e-prescription and an e-health call center). In the intervention, the patient had a Personal Health Folder, with clinical information, monthly telephone calls, a multidisciplinary team, and a personal plan drawn for each patient. Electronic messages between patients and/or carer and healthcare professional. A web-based education was accessible for patients in addition to face-to-face sessions with nurses. | **Communicate*  **Create a proactive plan of care  *Monitor, follow up   and respond to   change *Support self-  management goals* |
